# Insights Into Culturomics of the Rumen Microbiome

**DOI:** 10.3389/fmicb.2018.01999

**Published:** 2018-08-29

**Authors:** Tamar Zehavi, Maraike Probst, Itzhak Mizrahi

**Affiliations:** Institute of Natural Sciences, Department of Life Sciences, Ben-Gurion University of the Negev, Beersheba, Israel

**Keywords:** culturomics, gut microbial isolates, anaerobic microbiology, rumen microbiome, rumen microbiology, rare biosphere, cultivation

## Abstract

Cultivation of undescribed rumen microorganisms is one of the most important tasks in rumen microbiology. In this study, we aimed to discover the potential of culturomics for characterizing the rumen microbiome and for identifying factors, specifically sample dilution and media type, which affect microbial richness on agar plates. Our cultivation experiment captured 23% of all operational taxonomic units (OTUs) found in the rumen microbiome in this study. The use of different media increased the number of cultured OTUs by up to 40%. Sample dilution had the strongest effect on increasing richness on the plates, while abundance and phylogeny were the main factors determining cultivability of rumen microbes. Our findings from phylogenetic analysis of cultured OTUs on the lower branches of the phylogenetic tree suggest that multifactorial traits govern cultivability. Interestingly, most of our cultured OTUs belonged to the rare rumen biosphere. These cultured OTUs could not be detected in the rumen microbiome, even when we surveyed it across a 38 rumen microbiome samples. These findings add another unique dimension to the complexity of the rumen microbiome and suggest that a large number of different organisms can be cultured in a single cultivation effort.

## Introduction

The rumen microbiome enables ruminants to make energy stored in plant material metabolically available (Shabat et al., 2016). This achievement is the result of a complex web of interactions and energy flow among many different microorganisms, which is far from fully understood (Mizrahi, 2013). The composition of the rumen microbiome can influence the animal’s energy-harvesting ability, and the quality and quantity of specific microbial groups may tilt the scale in defining efficient vs. inefficient animals (Guan et al., 2008; Shabat et al., 2016; Sasson et al., 2017). In addition, different diets can impact the host animal by changing the rumen microbial composition and consequently, its functioning (Kittelmann et al., 2014; Friedman et al., 2017a,b). Understanding the complex interplay of the environment, microbiome, and animal host not only provides potential applicative benefits, but allows for deeper insights into microbial ecology.

This understanding implies potential answers to seemingly simple questions, such as: “Who is there?” and “What do they do?” In the past few decades, the scientific community has transformed its methodology for studying microbial communities: boosting high-throughput omics approaches, such as sequencing of metagenomes and metatranscriptomes, proteomics and large-scale metabolomics, have provided deeper insights and more available data (Yáñez-Ruiz et al., 2010; Morgavi et al., 2015; Wallace et al., 2015; Weimer et al., 2015; Huws et al., 2016). Despite these contributions, the various reference databases, which originate from microbial isolates, are still insufficient, limiting our ability to interpret and understand existing and new data, as well as our ability to experiment and recapitulate existing microbiome phenotypes. With the desire to fully understand how gut systems work synergistically and how the microbiome benefits the host, there is an urgent need to improve the reference databases and to isolate as-yet uncultured microorganisms. Therefore, in 2011, a Rumen Microbial Genomics (RGM) network was established. In addition, the “Hungate1000” project (Grigoriev et al., 2012; Seshadri et al., 2018) was initiated, aiming to increase the microbial culture collection of rumen origin. Currently, the “Hungate1000” catalog contains 410 genomes of rumen microorganisms, including different strains^1^. However, despite all of this effort, from the rumen environment only 3.6% of the operational taxonomic units (OTUs) found by sequencing have representative isolates (61 out of 1,698 OTUs) (Nordberg et al., 2014; Seshadri et al., 2018) in the RDP database (Cole et al., 2014), and only 117 bacterial species (not including different strains) of rumen origin are available from international culture collections: American Type Culture Collection (ATCC), Culture Collection University of Göteborg (CCUG), Leibniz Institute DSMZ-German Collection of Microorganisms and Cell Culture (DSMZ), Japan Collection of Microorganisms (JCM), the Belgian Co-Ordinated Collections of Microorganisms (BCCM/LMG), and the National Collection of Type Cultures (NCTC).

**FIGURE 1:**
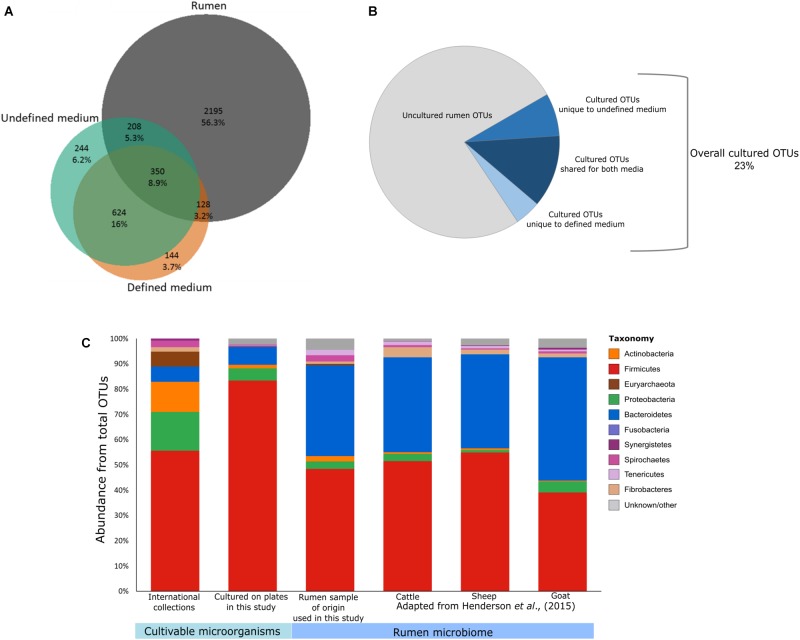
Potential of culturomics for the rumen microbiome. **(A)** Venn diagram illustrating the OTUs detected in the defined medium (orange), the undefined medium (green), and the original rumen sample and its dilutions (gray). Percentages were calculated from the sum of all samples. **(B)** Pie chart illustrating the cultivable and uncultivable fractions of the detected rumen microbiome. **(C)** Stacked bar plots describing the phylogenetic composition of available cultures from public collections (ATCC, CCUG, DSMZ, JCM, NCTC, and BCCM/LMG), the cultured OTUs detected on the plates, the rumen sample used for culturing in this study, and rumen microbiomes as reported previously by Henderson et al. (2015) (cattle, goat, and sheep).

Furthermore, most of the cultures are not available in every collection, with 88 out of 117 different species being found in any one of the six international collections, and the DSMZ having the highest number of cultures available. Moreover, the composition of the available cultures does not represent the phylogenetic composition of the rumen (**Figure 1A**). Hence, there is still a great need to increase the number of representative isolates.

The growth conditions applied in the laboratory are the most important aspect of isolating and cultivating microorganisms. With Robert E. Hungate’s development of anaerobic culturing methods (Chung and Bryant, 1997), the use of rumen fluid in growth media to isolate rumen microorganisms became the most common approach (Bryant and Burkey, 1953; Bryant and Small, 1956; Smith and Hungate, 1958; Bladen et al., 1961; Bryant and Robinson, 1961), even though “habitat-simulating” rumen fluids can vary among animals (Caldwell and Bryant, 1966) and even within the same individual following the consumption of different diets (Friedman et al., 2017b). Eventually, the transition from undefined to defined media became necessary, allowing for comparisons and reproducibility of results, and enabling different laboratories to grow the same isolates. Nonetheless, we believe that in addition to defined media, the initial use of rumen fluid for isolating as-yet uncultured rumen microorganisms can be beneficial. After isolation and characterization, the transition can be made to defined media for general availability.

Here, we aimed to (i) estimate the portion of the rumen microbiome that can be cultivated on either defined or undefined media and (ii) evaluate the effect of medium type and dilution on the overall cultivability of rumen microbes. In addition, we addressed the fraction of as-yet uncultured rumen microorganisms that can be cultured according to our results in a database-comparative manner. We hypothesized that using undefined medium would enable a higher number of different microorganisms to grow on the plates since the rumen fluid in the medium can act as a stimulating factor, making the medium compositionally more similar to the actual rumen. Diluting environmental samples prior to cultivation is a well-known method used to count cells and isolate microorganisms. Here, we sought to examine the effect of this method on our ability to culture both abundant and rare rumen microorganisms.

## Materials and Methods

### Animal Handling and Sampling

The experimental procedures in this study were approved by the Animal Policy and Welfare Committee of the Agricultural Research Organization Volcani Research Center and were in accordance with the guidelines of the Israel Council for Animal Care.

Israeli Holstein cows (*n* = 39) were fed a standard lactating cow diet *ad libitum* consisting of 30% roughage and 70% concentrates, and they had free access to water. The cows were sampled via the mouth using a stomach tube with a rumen vacuum sampler 1 h after the morning feeding. One rumen sample was transferred to two centrifuge tubes, one of which contained glycerol (10% final concentration). All rumen samples were kept at -20°C for 2 weeks until use.

### Medium Preparation

Medium 10 (M10) (Caldwell and Bryant, 1966) was used to grow rumen microorganisms. Slight modifications were made and the final medium was composed of (per 100 mL distilled water): 0.2 g trypticase, 0.05 g yeast extract, 3.8 mL solution of K_2_HPO_4_⋅3H_2_O (1.57 g in 200 mL distilled water), 3.8 mL salt solution [0.32 g CaCl_2_⋅2H_2_O, 12 g KH_2_PO_4_, 2.4 g NaCl, 1.2 g (NH_4_)_2_SO_4_, 0.5 g MgSO_4_⋅7H_2_O in 200 mL distilled water], 100 μL Hemin solution (1 g mL^-1^), 100 μL Resazurin solution (1 mg mL^-1^), 0.4 g NaHCO_3_, 0.1 g L-cysteine HCl, 100 μL of 1 M complex sugar mix (arabinose, xylose, glucose, galactose, cellobiose, sucrose, lactose, mannose, maltose, and rhamnose), 1 mL vitamin mix (as described in DSMZ medium 141) and 1.5 g agar. Additionally, 22.85 μL amphotericin B (2.5 mg mL^-1^) was added to prevent rumen fungi from growing on the plates. Amphotericin B is not known to affect bacterial growth. For the defined medium, 310 μL volatile fatty acid mix (Caldwell and Bryant, 1966) was added. For the undefined medium, we added 30 mL of clarified rumen fluid prepared in advance as follows: 1 L of frozen rumen fluid was thawed in a vinyl anaerobic chamber (COY Lab Products, Grass Lake, MI, United States) for 24 h, incubated with 4 g yeast extract at 39°C for 24 h and subsequently centrifuged in closed anaerobic centrifuge bottles at 13,000 *g*, 25°C for 25 min. The supernatant was autoclaved in the anaerobic bottles for 20 min at 121°C.

### Culturing of Rumen Microorganisms

A small fraction of the single rumen sample stored in glycerol was thawed on ice inside the anaerobic chamber. The anaerobic environment in the chamber was: 5% H_2_, 20% CO_2_, and 75% N_2_. The rumen sample was diluted in decimal format up to 10^-6^ using 1× anaerobic PBS which was filtered using a pore size of 0.22 μm (Merck Millipore Ltd., Tullagreen, County Cork, Ireland). A 100 μL aliquot of serially diluted rumen sample was plated in duplicate on each agar medium. The plates were incubated at 39°C inside the anaerobic chamber for 3 days, 24 plates overall. As a control, 2 plates from each media type were not plated but were incubated with the other plates. Additionally, we plated all buffers that were used for the dilutions of the rumen samples. After incubation, 1 mL of sterile anaerobic 0.85% NaCl solution was spread on each plate and used to collect all microbes from that plate by mechanically scraping and collecting the cells with a pipette.

### DNA Extraction

The rumen samples were separated according to Stevenson and Weimer (2007). The rumen samples (*n* = 39), rumen dilutions of the selected cow as inoculum source (*n* = 6) and microbial consortia from the plates (*n* = 24) were treated as previously described for DNA extractions (Stevenson and Weimer, 2007) with some modifications according to Jami et al. (2013) to suit the needs of this experiment. Briefly, 700 μl of each sample was mechanically broken up using a bead-beater. The DNA was separated from protein twice by adding phenol. Phenol was gradually removed from the DNA sample twice using phenol:chloroform (1:1). Subsequently, the samples were washed twice with chloroform. The DNA was precipitated in NaAc and isopropanol overnight at -20°C. The pellet was washed with ethanol and finally resuspended in TE. Overall, 24 plate samples, 7 rumen samples from the cultivation experiment, and 38 rumen samples were analyzed. The extraction of DNA was performed for all samples at the same day using the same materials and equipment.

### 16S Ribosomal DNA Sequencing

The V4 region of 16S rDNA was amplified by PCR from 69 DNA extracts using barcoded primers 515F 5′-CCTACGGGAGGCAGCAG-3′ and 806rcbR 5′-CCGTCAATTCMTTTRAGT-3′ (Peterson et al., 2009). The barcoded samples were pooled, sequenced in a MiSeq flow cell (Illumina) for 251 cycles from one end of the fragment and analyzed with Casava 1.8. Overall 1,366,443 reads were obtained for all samples, with an average of 16,663 ± 7,561 reads per sample.

### Sequence Data Analysis

Sequence data quality control and analyses were performed using the QIIME pipeline, version 1.7.0 (Caporaso et al., 2010). Briefly, the reads were demultiplexed into samples according to the different barcodes. Illumina adapters and primers were removed and the forward and reverse reads were paired. Sequences with barcode mismatches >1.5 or N bases were removed from the dataset, sequences were truncated prior to three bases with a low quality score (<3) and sequences <191 bp were excluded from further analyses. The average sequence size was 253 bp. OTUs were picked at 97% identity using UCLUST (Edgar, 2010). A *de novo* picking algorithm was applied in order to not exclude sequences from our dataset solely because of missing reference sequences. Taxonomic assignment of OTUs to the species level was performed by using RDP algorithm on the most abundant sequence of each OTU and the 16S rRNA reference database Greengenes (DeSantis et al., 2006). Assuming they were mainly chimeric or artifacts and in order to minimize random contaminations, singletons and doubletons were removed from the dataset. Data subsampling was performed according to the sample with the least amount of reads (8,078 sequences) and an OTU table was generated, containing the number of reads for each OTU in each sample. In addition, an OTU table containing relative abundances of non-subsampled reads was produced. Sequences were deposited to the SRA under the accession number SRP153190.

### Diversity and Similarity

To assess the similarity between microbial consortia that grew on the plates to both each other and the original rumen dilution, principal coordinates analysis (PCoA) was performed on the subsampled OTU table of rumen samples (rumen sample of origin) and all the microbes collected from each plate. Distances were calculated using Euclidean distance. Eigenvalues were calculated for each axis. The significance of the dilution factor and medium type was calculated using two-way PERMANOVA on 1,000 permutations. Sum of squares was utilized to calculate the variance explained by the factors. A confidence interval of 95% was applied.

### Richness Assessment

To assess the ability and reproducibility of the cultivation conditions used in the experiment to support growth of a certain number of different OTUs, the richness (observed OTUs) was examined in each sample based on two technical repetitions. The richness was then compared between different dilutions of the same rumen sample using Wilcoxon rank sum test.

### Proportion of Shared OTUs

The proportion of microorganisms that are potentially cultivable using this method was estimated from the non-subsampled OTU table. The proportions of shared OTUs were calculated between the rumen samples and their matching plates per dilution, as well as the overall shared OTUs between the datasets (rumen samples, microbial consortia from defined medium and microbial consortia from undefined medium). Venn diagrams were created using the Shiny web application for Rstudio, eulerr online tool^2^.

### Phylogenetic Distances and Tree Construction

The phylogenetic variety found on the different media and in the original rumen sample, including its plated dilutions, was compared using the average pairwise phylogenetic distance between OTUs. Therefore, a fasta file listing the sequences of all OTUs from the rumen (both cultivable and uncultivable) and a file listing all cultivable OTUs were created. A distribution of the average phylogenetic distances of randomly selected OTUs from the rumen was created (1,000 OTUs, 100 permutations). The average phylogenetic distance between cultivable OTUs was calculated and *P*-values were obtained using the random distribution of OTUs from the rumen.

To measure the average phylogenetic distances of OTUs within a specific taxonomic group (family level), a fasta file of all OTUs belonging to each family was generated and the permutation test was repeated. This analysis was performed for families with at least 10 OTUs in both the rumen sample of origin (including the different dilutions) and the cultured OTUs.

**FIGURE 2:**
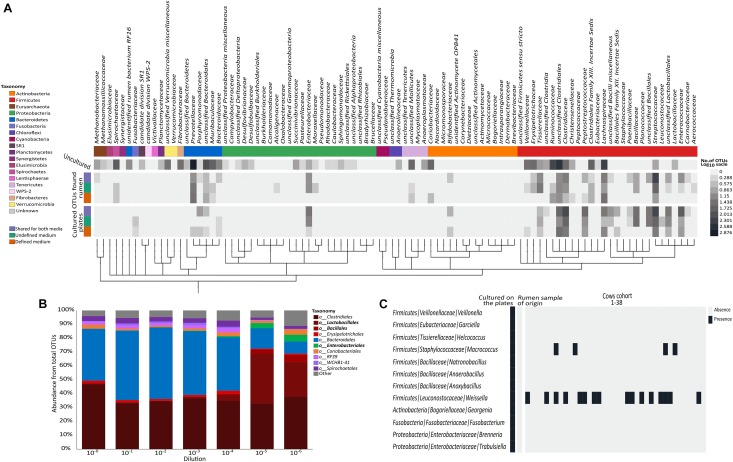
Distribution of the cultured OTUs across rumen microbiome taxonomy and structure. **(A)** Heat map describing the number of OTUs (in log scale) annotated to phylogenetic groups detected in each of the examined datasets. From top to bottom (color coding indicated on the left side of the figure): OTUs detected only in the original rumen sample, OTUs detected on both media and in the original rumen sample, OTUs detected on the undefined medium and in the rumen sample, OTUs detected on the defined medium and in the rumen sample, OTUs detected on both media, OTUs detected only on the undefined medium, and OTUs detected only on the defined medium. OTUs were arranged on a phylogenetic tree that was created by phyloT program using NCBI accession numbers for each phylogenetic group. The tree was visualized using iTOL online tool (Letunic and Bork, 2016). **(B)** Stacked bar plot describing the relative abundance at the order level of the original rumen sample and its dilutions. **(C)** Heat map describing the distribution of the cultured OTUs belonging to the rare biosphere across 38 rumen microbiomes. Taxonomic annotations are given as phylum/family/genus.

To create a general overview of the microorganisms detected in our dataset (**Figure 2A**), we searched NCBI accession numbers at the family level or higher (Sayers et al., 2009; Benson et al., 2010) and used the phyloT online tool^3^ to create distances between microbial families. Taxonomic phylum level and a heat map representing the log number of OTUs found in each group (unique to plates per medium, cultivable OTUs found in the microbiome per medium and uncultivable OTUs) were added. The data were visualized using the iTOL online tool (Letunic and Bork, 2016).

### Comparison to “Hungate1000” Database

We used the “Hungate1000” database downloaded on June 5th 2018. The cultured OTUs from our dataset were blasted against this “Hungate1000” database (*e*-value = 0.005) (Seshadri et al., 2018). Results and taxonomy were tested for OTU hits of 100% similarity and 97–99.9% similarity separately. The “Hungate1000” database contains 410 genomes of rumen microorganisms (including different strains).

## Results and Discussion

### Most Cultivable Rumen Microbes Belong to the Rumen Rare Biosphere

For the purpose of isolating as-yet uncultured rumen microbes, it is important to identify which factors influence their cultivability. Here we focused on the effects of phylogenetic association, medium type, and sample dilution. We plated several serial dilutions (10^-1^–10^-6^) of a cow’s rumen sample on both defined medium (M10) and undefined medium (M10 supplemented with rumen fluid). We then subjected all microbes growing on the plates to 16S rDNA gene amplicon sequencing. In addition, we sequenced the original rumen sample and the serial dilutions (up to 10^-6^) that were used for plating, in order to capture which OTUs grew on the plates in comparison to what had been plated. Using the resulting dataset, we first examined the effect of media type on the cultivability by comparing the microbial composition growing on defined and undefined media. The undefined medium containing rumen fluid increased the cultured richness by a factor of two compared to the defined medium (**Figures 1A,B**). This suggests that the more natural conditions of the rumen fluid provide unidentified growth-promoting factors that enable a greater variety of OTUs to grow. Nevertheless, we found 272 specific OTUs on the defined medium as well. Therefore, utilization of both defined and undefined media can increase the number of different cultured OTUs by up to 40%. It should be noted that the sample plated had been frozen in 10% glycerol. Therefore, microbes sensitive to the freezing and thawing would not have been cultivated and thus, these numbers might increase for freshly collected samples.

Out of the total 2,881 OTUs detected in the original rumen sample and its dilutions, 686 OTUs were also found on the plates. This suggests that 23% of the rumen microbiome is potentially cultivable. These cultured OTUs belonged to at least 10 bacterial and 1 archaeal phyla (**Supplementary Table S1**). We found a total of 82 bacterial genera, 58 of which did not have isolated representatives in any of the international microbial collections (ATCC, CCUG, DSMZ, JCM, NCTC, or BCCM/LMG) according to our survey. Only 45 isolates in these culture collections corresponded by name to OTUs from the plates that had species level annotation in our dataset. Only 5 of these representative species had been isolated from bovine or sheep, and the rest originated from various environments (**Supplementary Tables S2**, **S3**). As the “Hungate1000” consolidates most of the rumen isolates known to date (Jun 5, 2018), we compared the OTUs found on the plates to this reference (Seshadri et al., 2018). We found a very small overlap between the “Hungate1000” database and the OTUs from our cultivation experiment (61 out of 1,698 cultivable OTUs in our study). This small overlap highlights the importance of international efforts to capture cultivable rumen diversity while it also emphasizes the enormous potential to increase the diversity of rumen culture collections, even in a single cultivation effort.

Sequencing the plated sample dilutions provided deeper insight into the complexity of the rumen microbiome as provided by sequencing the original rumen sample. With increasing dilution of the rumen sample, the percentage of reads annotated as *Bacteroidetes* strongly decreased, while more and more reads belonged to the phylum *Firmicutes* (**Figure 2B** and **Supplementary Figure S1**). At the order level, higher numbers of *Lactobacilli, Bacilli*, and *Enterobacteria* were found with increasing dilution of the rumen sample (**Figure 2B**), which generally fit the phylogenetic composition of microbes that grew on the plates (**Figure 1C**).

Interestingly, we found that most of the OTUs that grew on the plates (1,012 out of 1,698) were not detected in the original rumen sample or any of its dilutions (**Figure 1A**). Some of these 1,012 OTUs belonged to various lineages up to the phylum level that were not found in the original rumen samples (**Figure 2C**). Most of the microbes that were cultured on the plates remained undetectable, even in the extremely diluted rumen samples. With an average read number of 21,975 ± 5,847 the sequencing effort was close to saturation in individual rarefaction curves (**Supplementary Figure S2**). As we could exclude systematic contaminations by plating the buffers that were used for the dilutions of the rumen sample as well as by incubating the plates themselves, this finding lead us to hypothesize that the microbes cultured in our experiment might be affiliated with the rare portion of the rumen biosphere. This hypothesis was supported by the large number of OTUs which were unique to the plates. Although random contaminations can never be completely excluded, in combination with the empty control plates, it is unlikely that our finding is a result of random contaminations. Excluding contaminations as a cause for the observation, we further explored the presence of these microbes in multiple rumen ecosystems by sequencing 38 additional cow rumen microbiomes. Except for the genera *Weissella* and *Macrococcus* of the phylum *Firmicutes*, most of these extremely rare biosphere microbes could not be found in this large number of samples (**Figure 2C**). In multiple microbial ecosystems, including freshwater (Musat et al., 2008), sludge (Hernandez-Raquet et al., 2013), soil (Pester et al., 2010; Giebler et al., 2013; Philippot et al., 2013), rhizosphere (Nuccio et al., 2016), and human microbiome (Guss et al., 2011), the rare microbiome is now being acknowledged as highly influential in terms of ecosystem functions and hosts’ health. In these studies, the rare microbes are reported to affect their environment via multiple mechanisms such as enhancing functionality of abundant microbes, being more active than the abundant microbes, hampering the invasion of other species (Mallon et al., 2015) during community assembly and contributing to the metabolic potential of the community by increasing the genetic pool of their environment (Lynch and Neufeld, 2015; Jousset et al., 2017). Thus, rumen complexity seems to be even higher than previously thought, and the rare microbes of this ecosystem are not necessarily uncultivable. The abundance of these rare OTUs might vary as a function of environmental conditions. Consequently, isolating and studying these organisms becomes even more relevant given that they could have a tremendous impact on the ecosystem.

### Phylogenetic Distances Between Cultured OTUs on the Lower Tree Branches Suggest That Multifactorial Traits Determine Cultivability

**FIGURE 3:**
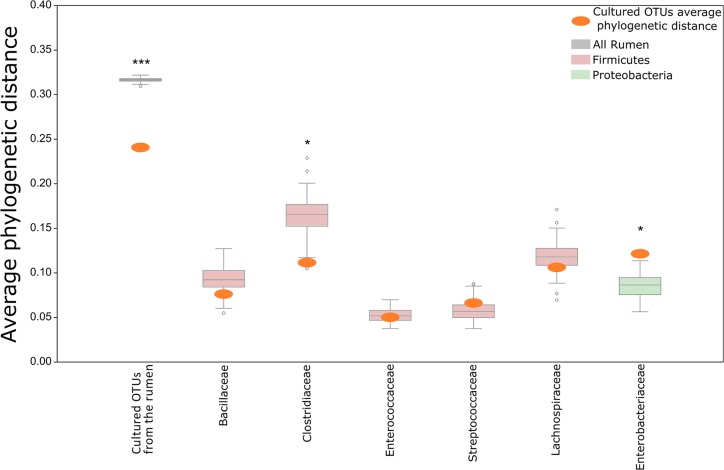
Comparison of the phylogenetic distances between OTUs growing on the plates and OTUs detected in the original rumen sample. The mean phylogenetic distances between OTUs that grew on plates was calculated and compared to the distribution of phylogenetic distances between the OTUs of the original rumen sample. The distribution of distances from the rumen OTUs is illustrated as a box plot. The average phylogenetic distance between OTUs of the cultured reference group being compared to this rumen distribution is illustrated as an orange oval. The distribution of phylogenetic distances was calculated from a rumen OTU subset picked randomly in a sample size depending on the cultured OTU reference group. Permutation = 100. The following numbers of rumen OTUs were picked for each category (according to the *x*-axis): All = 1,012; *Bacillaceae* = 6, *Clostridiaceae* = 6, *Enterococcaceae* = 6, *Lachnospiraceae* = 6, *Streptococcaceae* = 6, *Enterobacteriaceae* = 6. Asterisks mark in which cases the average phylogenetic distance of the cultured OTUs differs from the average distance of uncultured OTUs. ^∗^Cultured OTUs are above/below the 10th percentile. ^∗∗∗^Cultured OTUs are below the 1st percentile.

Current culture collections do not reflect a typical rumen composition (**Figure 1C**; Jami and Mizrahi, 2012; Henderson et al., 2015). Moreover, our findings show that there is a discrepancy in taxonomic composition between cultured OTUs and the rumen microbiome (**Figure 1C**). Nevertheless, almost all rumen microbiome phyla were represented within the cultured OTUs, and *Firmicutes* and *Bacteroidetes* made up 90% of the cultured OTUs on the plates (**Figures 1C**, **2A**), which is comparable to the occupancy of these phyla in the original rumen sample (**Figure 1C**). This was surprising since the public collections ATCC, CCUG, DSMZ, JCM, NCTC, and BCCM/LMG have a lower fit to the phylogenetic distribution of a typical ruminant’s microbiome (**Figure 1C**) according to our survey, as well as that conducted by Creevey et al. (2014). In these collections, *Proteobacteria* and *Actinobacteria* are highly overrepresented, whereas *Bacteroidetes* is underrepresented (Creevey et al., 2014). *Bacteroidetes*, e.g., is represented by only 7 known isolates in international collections, whereas we cultured 121 OTUs belonging to six different families (**Supplementary Table S1**). This suggests that cultivability might be connected to the phylogenetic association of some microbial groups. If this is true, the cultured OTUs might show a phylogenetic cohesiveness that could differentiate them from the uncultured ones. With this in mind, we tested the average phylogenetic distances of OTUs cultured on the plates against randomly selected OTUs from the rumen microbiome of the original sample (**Figure 3** and **Supplementary Figure S3**). The OTUs that were found on the plates were more closely related to each other compared to those detected in the original rumen sample (**Figure 3**). This supports our hypothesis that the cultured OTUs are more phylogenetically cohesive and suggests that certain genetic traits are involved in the ability to grow on plates. Indeed, the main proportion of the cultured OTUs was annotated as *Firmicutes* (**Figure 3**) which is in agreement with Creevey et al. (2014), who reported that this phylum is the most represented in cultured isolates. The 1,415 *Firmicutes* OTUs present in our dataset were distributed across 50 genera (**Supplementary Table S1**), whereas isolates from 45 genera are available in public culture collections.

We then asked whether this assumption holds true on a finer taxonomic level. We examined OTUs from the most abundant families on the plates (>10 OTUs per family) and compared them to randomly selected OTUs from the same family coming from the original rumen microbiome. The decreased phylogenetic distance was not consistent across the tested phylogenetic families. For the *Firmicutes* families *Bacillaceae* and *Clostridiaceae*, respectively, we detected smaller phylogenetic distances (*P_Bacillaceae_* = 0.1; *P_Clostridiaceae_* < 0.05) between the OTUs on the plates and those in the original rumen sample. In contrast, there was a higher phylogenetic distance between *Proteobacteria* OTUs annotated as *Enterobacteriaceae* from the plates and the *Enterobacteriaceae* OTUs from the original rumen sample (*P* < 0.01). These findings suggest that cultivability of microbes on the plates is a multifactorial trait, selecting different clades that are scattered across the branches of the phylogenetic tree rather than being confined to a specific clade.

### Sample Dilutions and Different Media Increase Cultivability

**FIGURE 4:**
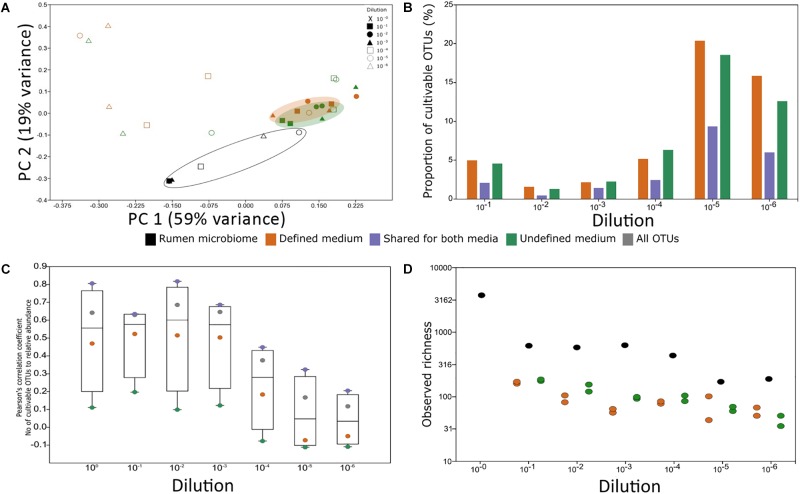
Experimental factors affecting cultivability. **(A)** PCoA of rumen samples and their cultivable microbial consortia. The variance explained by each component is indicated on each axis. **(B)** Proportion of the cultivable microbiome in each rumen dilution. The proportion was calculated as the number of OTUs found in both the rumen dilution and its matching plate. The *x*-axis denotes the dilution of the sample. There was no statistical difference between the proportions of OTUs growing on defined and undefined media (Wilcoxon rank sum test, *P* > 0.05). **(C)** The impact of abundance on cultivability. Pearson’s correlation coefficient of the number of cultivable OTUs in each percentile and the relative abundance in each percentile was calculated. Correlations above | *r*| = 0.3 and *P* < 0.05 after Holm–Bonferroni correction were considered significant. The *y*-axis represents Pearson’s correlation coefficient result and the x axis represents the dilution. The numbers of cultivable OTUs were divided according to the medium in which they were found (green – OTUs unique to undefined medium, orange – OTUs unique to defined medium, purple – OTUs found on both medium types, and gray – total number of OTUs). **(D)** Observed richness for rumen samples and microbial consortia from the plates in each medium type. The overall difference between groups was significant as determined by Kruskal–Wallis test (*P* = 0.01564). Wilcoxon rank sum paired test (*P*-value Holm–Bonferroni correction): rumen vs. defined medium: *P* = 0.03125; rumen vs. undefined medium: *P* = 0.03125 from each plate in each medium type. Colors represent sample source: Black – rumen samples, orange – microbial consortium that grew on defined medium, and green – microbial consortium that grew on undefined medium. *X*-axis denotes the dilution of the sample.

To understand which factor, i.e., medium type or dilution, has a stronger effect on our ability to cultivate rumen microorganisms, PCoA based on a Euclidean distance matrix was applied (**Figure 4A**). Independent of their origin (plate cultivation or original rumen sample), coordinate 1 separated all consortia by the dilution factor, which explained 30% of the variance in the dataset. It should be noted that depending on the dilution, each plate contained a comparable number of OTUs (100–300). However, with increasing dilution, the compositional similarity between the plates and also between technical repetitions of the same dilution diverged (**Figure 4A** and **Supplementary Figure S4**; see also **Figure 1**). This phenomenon potentially stems from the random sampling of this rare biosphere during the process of diluting and plating, which resulted in a distinct microbial composition on each plate.

In reduced dimensionality, there was no difference in the microbial composition that grew on the defined and undefined media, independent of dilution. However, underlining the phylogenetic differences between available cultures and a typical ruminant’s microbiome, there was a clear difference between the rumen microbiome and the microbes cultured on the plates. The factor origin, i.e., rumen or medium, explained 18% of the dataset’s variance (**Figure 4A**). Based on these analyses, it is mainly dilutions and repetitions that allow for different microbes to grow on the plates and likely also for a higher microbial diversity to be captured by cultivation (**Figures 4A,B**).

### OTU Abundance in the Rumen Environment Is Positively Correlated to Cultivability

We next sought to understand whether there is a connection between cultivability of an OTU and its abundance. Overall, the total number of cultured OTUs was positively and significantly correlated to their relative abundance in the less diluted rumen samples (**Figure 4C**). The number of cultured OTUs from highly diluted samples was less dependent on the relative abundance of OTUs in the sample (**Figure 4C**), suggesting that sample dilution is highly relevant to reducing the dependence of cultivability on abundance. While the number of cultured OTUs unique to the defined medium was correlated to their abundance, this dependence was practically non-existent for the undefined medium. However, OTUs which were found on both media were highly dependent on the relative abundance of the corresponding microorganisms in the sample (**Figure 4C**). This emphasizes the potential of using rumen fluid in the culture medium. These findings suggest that factors like sampling time and method, related to the abundance of the different rumen microbiome members, may affect the composition of cultivable microbes. For example, the rumen microbiome undergoes compositional diurnal oscillations (Shaani et al., 2018) which in turn may affect the probability of the different microbes to be sampled and subsequently cultivated.

Using sample dilutions, the culture conditions allowed growth of both the highly abundant and eminently low abundant OTUs (**Figure 4C**) and enabled capturing the highest proportion of cultured OTUs (**Figure 4C**). This is in agreement with studies on the human gut microbiome (Walker et al., 2014; Lagkouvardos et al., 2017). Thus, the isolation of rumen microorganisms should become one of the major focuses of our field, to assign better annotations and to better understand the rumen ecosystem.

## Conclusion

In this study, we asked two fundamental questions: (i) What is the portion of the rumen microbiome that can potentially be cultivated? (ii) How are medium type, sample dilution and phylogeny related to cultivability? To answer these questions, we used defined and undefined (with rumen fluid) anaerobic media and plated decimal dilutions of a rumen sample. Using this methodology, 23% of the rumen microbiome is potentially cultivable. We found a positive correlation between the cultivability of an OTU and its abundance in the original rumen sample for both media. The effect of sample dilution exceeded the effect of medium type, indicating that the laborious plating effort is worth the chance of capturing rumen microbial diversity. Furthermore, technical replications in plating strongly contributed to the richness on the plates and both media had a high number of unique OTUs. Cultivation seems to select for a multifactorial set of genetic traits scattered across the phylogenetic tree. Despite the great variety of microbes cultured on the plates, the selection for genomic traits also calls for new isolation approaches based on certain sets of traits to increase cultivated diversity, as these genetic traits are not necessarily interconnected and unlikely to be fully dependent on each other.

The OTUs cultured here were mainly from the rare rumen biosphere. Taken together, the high relevance of the rare biosphere reported in the literature and its surprisingly high cultivability as found here, we conclude that the rumen’s complexity and functional repertoire are beyond our current estimation. Nonetheless part of these rare biosphere microbes can be studied in pure culture, enabling a deeper understanding of rumen ecosystem functionality. Therefore, our study presents a very promising notion: a vast variety of unknown rumen microorganisms can be grown using defined and undefined anaerobic media.

## Author Contributions

TZ designed the experiments, collected the samples, carried out the wet lab experiments, designed and performed the analyses, and wrote the manuscript. MP contributed to data analyses and wrote the manuscript. IM designed the experiments, designed the data analysis, and wrote the manuscript.

## Conflict of Interest Statement

The authors declare that the research was conducted in the absence of any commercial or financial relationships that could be construed as a potential conflict of interest.

## Footnotes

1 https://genome.jgi.doe.gov/portal/TheHunmicrobiome/TheHunmicrobiome.info.html

2 https://jolars.shinyapps.io/eulerr/

3 http://phylot.biobyte.de/
