# Supplementary material for: Insights Into Culturomics of the Rumen Microbiome
Source: Front Microbiol. 2018 Aug 29;9:1999. doi: 10.3389/fmicb.2018.01999 (PMC6123358; doi:10.3389/fmicb.2018.01999)
Supplement: Supplementary file 1 [file Table_1.PDF]

Table S1. Number of different annotations per phylogenetic level idetified in this study for cultivable OTUs
*only annotated OTUs were counted at each phylogenetic level seperatly

	Phyla	Number of OTUs	Number of different classes	Number of different orders	Number of different families	Number of different genera	Number of different species
**Bacteria**	Actinobacteria	24	2	3	6	5	4
	Bacteroidetes	121	1	1	7	6	3
	Cyanobacteria	2	2	2			
	Firmicutes	1415	3	5	22	50	30
	Fusobacteria	6	1	1	1	1	
	Proteobacteria	82	4	6	9	16	8
	Spirochaetes	7	1	1	1	1	
	Synergistetes	1	1	1	1	1	
	Tenericutes	5	1	1			
	Verrucomicrobia	1	1	1	1		
	Unknown	32					
**Archaea**	Euryarchaeota	3	2	2	2	2	
