# Supplementary material for: Insights Into Culturomics of the Rumen Microbiome
Source: Front Microbiol. 2018 Aug 29;9:1999. doi: 10.3389/fmicb.2018.01999 (PMC6123358; doi:10.3389/fmicb.2018.01999)
Supplement: Supplementary file 2 [file Table_2.pdf]

Table S2. List of genera with reported cultured rumen isolates and not reported but identified in this study
* marked detected in the rumen only for genera detected as cultivable in our study

Phylum	Rumen Bacterial Genera	Reported cultured isolate (Creevey et al.)	Identified in this study	Detected in Rumen sample (*)
Actinobacteria	Actinomyces	+	+	+
Actinobacteria	Atopobium	+	-	-
Actinobacteria	Bifidobacterium	+	+	+
Actinobacteria	Cellulomonas	+	-	-
Actinobacteria	Corynebacterium	+	+	+
Actinobacteria	Denitrobacterium	+	-	-
Actinobacteria	Georgenia	-	+	-
Actinobacteria	Micrococcus	-	+	+
Actinobacteria	Micromonospora	+	-	-
Actinobacteria	Olsenella	+	-	-
Actinobacteria	Prausurella	+	-	-
Actinobacteria	Propionibacterium	+	-	-
Actinobacteria	Slackia	+	-	-
Bacteriodetes	5-7N15	-	+	-
Bacteriodetes	Bacteroides	+	+	+
Bacteriodetes	CF231	-	+	+
Bacteriodetes	Parabacteroides	-	+	+
Bacteriodetes	Porphyromonas	+	-	-
Bacteriodetes	Prevotella	+	+	+
Bacteriodetes	Ruminobacillus	+	-	-
Bacteriodetes	Ruminofilibacter	+	-	-
Bacteriodetes	Sphingobacterium	+	-	-
Bacteriodetes	YRC22	-	+	+
Fibrobacteres	Fibrobacter	+	-	-
Firmicutes	02d06	-	+	+
Firmicutes	Acetitomaculum	+	-	-
Firmicutes	Acidaminococcus	+	-	-
Firmicutes	Aerococcus	-	+	+
Firmicutes	Alkaliphilus	-	+	+
Firmicutes	Allisonella	+	-	-
Firmicutes	Anaerobacillus	-	+	-
Firmicutes	Anaerovibrio	+	+	+
Firmicutes	Anaerovorax	+	+	+
Firmicutes	Anoxybacillus	-	+	-
Firmicutes	Bacillus	+	+	+
Firmicutes	Blautia	+	+	+
Firmicutes	Butyrivibrio	+	+	+
Firmicutes	Cellulosilyticum	+	-	-
Firmicutes	Clostridium	+	+	+
Firmicutes	Coprococcus	+	+	+
Firmicutes	Desemzia	-	+	+
Firmicutes	Desulfotomaculum	+	-	-

Firmicutes	Dorea	-	+	+
Firmicutes	Enterococcus	+	+	+
Firmicutes	Eubacterium	+	+	+
Firmicutes	Exiguobacterium	-	+	+
Firmicutes	Facklamia	-	+	+
Firmicutes	Garciella	-	+	-
Firmicutes	Helcococcus	-	+	-
Firmicutes	Howardella	+	-	-
Firmicutes	Kandleria	+	-	-
Firmicutes	Lachnobacterium	+	-	-
Firmicutes	Lachnospira	+	-	-
Firmicutes	Lactobacillus	+	+	+
Firmicutes	Lactococcus	+	-	-
Firmicutes	Lentibacillus	-	+	+
Firmicutes	Leuconostoc	+	-	-
Firmicutes	Macrococcus	-	+	-
Firmicutes	Megasphaera	+	-	-
Firmicutes	Mitsuokella	+	-	-
Firmicutes	Mogibacterium	-	+	+
Firmicutes	Moryella	-	+	+
Firmicutes	Natronobacillus	-	+	-
Firmicutes	Oribacterium	+	-	-
Firmicutes	Oscillibacter	+	-	-
Firmicutes	Oxobacter	+	-	-
Firmicutes	p-75-a5	-	+	+
Firmicutes	Paenibacillus	+	+	+
Firmicutes	Pediococcus	+	-	-
Firmicutes	Peptoniphilus	+	-	-
Firmicutes	Peptostreptococcus	+	-	+
Firmicutes	Phascolarctobacterium	-	+	+
Firmicutes	Proteiniclasticum	+	+	+
Firmicutes	Pseudobutyrivibrio	+	+	+
Firmicutes	Quinella	+	-	-
Firmicutes	RFN20	-	+	+
Firmicutes	Roseburia	-	+	+
Firmicutes	Ruminococcus	+	+	-
Firmicutes	Saccharofermentans	+	-	-
Firmicutes	Sarcina	+	-	-
Firmicutes	Schwartzia	+	-	-
Firmicutes	Sedimentibacter	-	+	+
Firmicutes	Selenomonas	+	-	-
Firmicutes	Sharpea	+	+	+
Firmicutes	Shuttleworthia	-	+	+
Firmicutes	SMB53	-	+	+
Firmicutes	Sporanaerobacter	-	+	+
Firmicutes	Staphylococcus	+	+	+
Firmicutes	Streptococcus	+	+	+
Firmicutes	Succiniclasticum	+	+	+
Firmicutes	Syntrophococcus	+	-	-
Firmicutes	Tepidimicrobium	-	+	+
Firmicutes	Tetragenococcus	-	+	+

Firmicutes	Tissierella	+	+	+
Firmicutes	Trichococcus	-	+	+
Firmicutes	Turicibacter	-	+	+
Firmicutes	Veillonella	+	+	-
Firmicutes	Virgibacillus	-	+	+
Firmicutes	Weissella	-	+	-
Fusobacteria	Fusobacterium	+	+	-
Proteobacteria	Acinetobacter	+	+	+
Proteobacteria	Actinobacillus	+	-	-
Proteobacteria	Alysiella	+	-	-
Proteobacteria	Ancylobacter	+	-	-
Proteobacteria	Basfia	+	-	-
Proteobacteria	Brenneria	-	+	-
Proteobacteria	Campylobacter	+	+	-
Proteobacteria	Desulfovibrio	+	-	-
Proteobacteria	Enhydrobacter	-	+	+
Proteobacteria	Enterobacter	-	+	+
Proteobacteria	Erwinia	-	+	-
Proteobacteria	Escherichia	+	-	-
Proteobacteria	Janthinobacterium	-	+	+
Proteobacteria	Klebsiella	+	-	-
Proteobacteria	Lampropedia	+	-	-
Proteobacteria	Mannheimia	+	-	-
Proteobacteria	Oxalobacter	+	-	-
Proteobacteria	Paracoccus	-	+	+
Proteobacteria	Pelomonas	-	+	+
Proteobacteria	Pigmentiphaga	+	-	-
Proteobacteria	Proteus	+	-	-
Proteobacteria	Providencia	-	+	+
Proteobacteria	Pseudomonas	+	+	+
Proteobacteria	Ralstonia	-	+	+
Proteobacteria	Ruminobacter	+	+	+
Proteobacteria	Salmonella	-	+	+
Proteobacteria	Shigella	+	-	-
Proteobacteria	Succinimonas	+	-	-
Proteobacteria	Succinivibrio	+	-	-
Proteobacteria	Sutterella	-	+	+
Proteobacteria	Trabulsiella	-	+	-
Proteobacteria	Wolinella	+	-	-
Spirochaetes	Treponema	+	+	+
Synergistetes	Synergistes	+	-	-
Synergistetes	Pyramidobacter	-	+	+
Tenericutes	Anaeroplasma	+	-	-
Tenericutes	Asteroleplasma	+	-	-
**Archaea**				
Euryarchaeota	vadinCA11	Archeae not mentioned	+	+
Euryarchaeota	Methanobrevibacter	Archeae not mentioned	+	+
