# Supplementary material for: Insights Into Culturomics of the Rumen Microbiome
Source: Front Microbiol. 2018 Aug 29;9:1999. doi: 10.3389/fmicb.2018.01999 (PMC6123358; doi:10.3389/fmicb.2018.01999)
Supplement: Supplementary file 3 [file Table_3.PDF]

Table S3. List of cultivable species *indicated in Creevey, Kelly et al. 2014

Organism	Origin of reference isolate	Reference	Detected in our rumen sample
Acinetobacter johnsonii	Duodenum	ATCC 17909	+
Alkaliphilus transvaalensis	mine water	ATCC 700919	+
Bacillus asahii	soil	JCM 5838	+
Bacillus thermoamylovorans	compost reactor	DSM 28918	+
Bacteroides uniformis	human feces	ATCC 8492	
Bifidobacterium adolescentis	bovine	DSM 20087 (*)	
Bifidobacterium pseudolongum	rumen/bovine/rumen	DSM 20096, JCM7092, ATCC 25864 (*)	+
Blautia producta	human feces	DSM 2950	+
Clostridium butyricum	swine intestine	ATCC 19398	+
Clostridium intestinale	cat feces	ATCC 49213	
Clostridium neonatale	human blood	ATCC BAA-265	+
Clostridium subterminale	Anaerobic sewage sludge	ATCC 29748	
Clostridium tetani	sheep	ATCC 454	+
Clostridium thermopalmarium	Palm wine	ATCC 51427	+
Corynebacterium simulans	human lymph node	ATCC BAA-15	+
Enhydrobacter aerosaccus	fresh water	ATCC 27094	+
Enterobacter cowanii	Blood	JCM 10956	
Enterococcus asini	Donkey caecum	ATCC 700915	
Enterococcus casseliflavus	plant material	ATCC 25788	+
Enterococcus cecorum	Chicken caecum	ATCC 43198	+
Enterococcus haemoperoxidus	service water	ATCC BAA-382	
Erwinia oleae	Olive tree	DSM 23412	
Eubacterium cylindroides	human feces	ATCC 27805	+
Janthinobacterium lividum	soil	ATCC 14273	+
Lactobacillus agilis	Chicken/swine intestine	JCM 1048, BCM 1049	+
Micrococcus luteus	clinical isolate	ATCC 49732	+
Paenibacillus barengoltzii	Equipment	ATCC BAA-1209	+
Paenibacillus lautus	Human intestine	ATCC 43898	+
Paenibacillus lentimorbus	beetle	ATCC 14707	
Paenibacillus macerans	potato	ATCC 8509	+
Prevotella copri	human feces	DSM 18205	+
Prevotella ruminicola	bovine	ATCC 19189 (*)	+
Providencia stuartii	clinical isolate	ATCC 49809	
Pseudomonas veronii	mineral water	ATCC 700474	+
Roseburia faecis	human feces	DSM 16840	
Sharpea azabuensis	bovine	DSM 20406 (*)	+
Staphylococcus sciuri	squirrel skin	DSM 20345	+
Streptococcus agalactiae	milk	ATCC 13813	+
Streptococcus alactolyticus	swine intestine	ATCC 43077	
Streptococcus luteciae	human isolate	LMG 22271	+
Streptococcus minor	cat tonsil	LMG 14394	
Tetragenococcus halophilus	degraded sugar thick juice	DSM 23766	+
Trabulsiella farmeri (Citrobacter farmeri)	human feces	ATCC 51633	
Veillonella parvula	intestinal tract	DSM 2008	
Virgibacillus pantothenticus	Soil	ATCC 14576	+
