# Supplementary material for: Insights Into Culturomics of the Rumen Microbiome
Source: Front Microbiol. 2018 Aug 29;9:1999. doi: 10.3389/fmicb.2018.01999 (PMC6123358; doi:10.3389/fmicb.2018.01999)
Supplement: Supplementary file 4 [file Image_1.PDF]

Frontiers in Microbiology

Metaomics approaches to study the rumen microbiome: Challenges and innovation

Insights into the culturomics of the rumen microbiome

Tamar Zehavi, Maraike Probst, Itzhak Mizrahi

Supplementary information

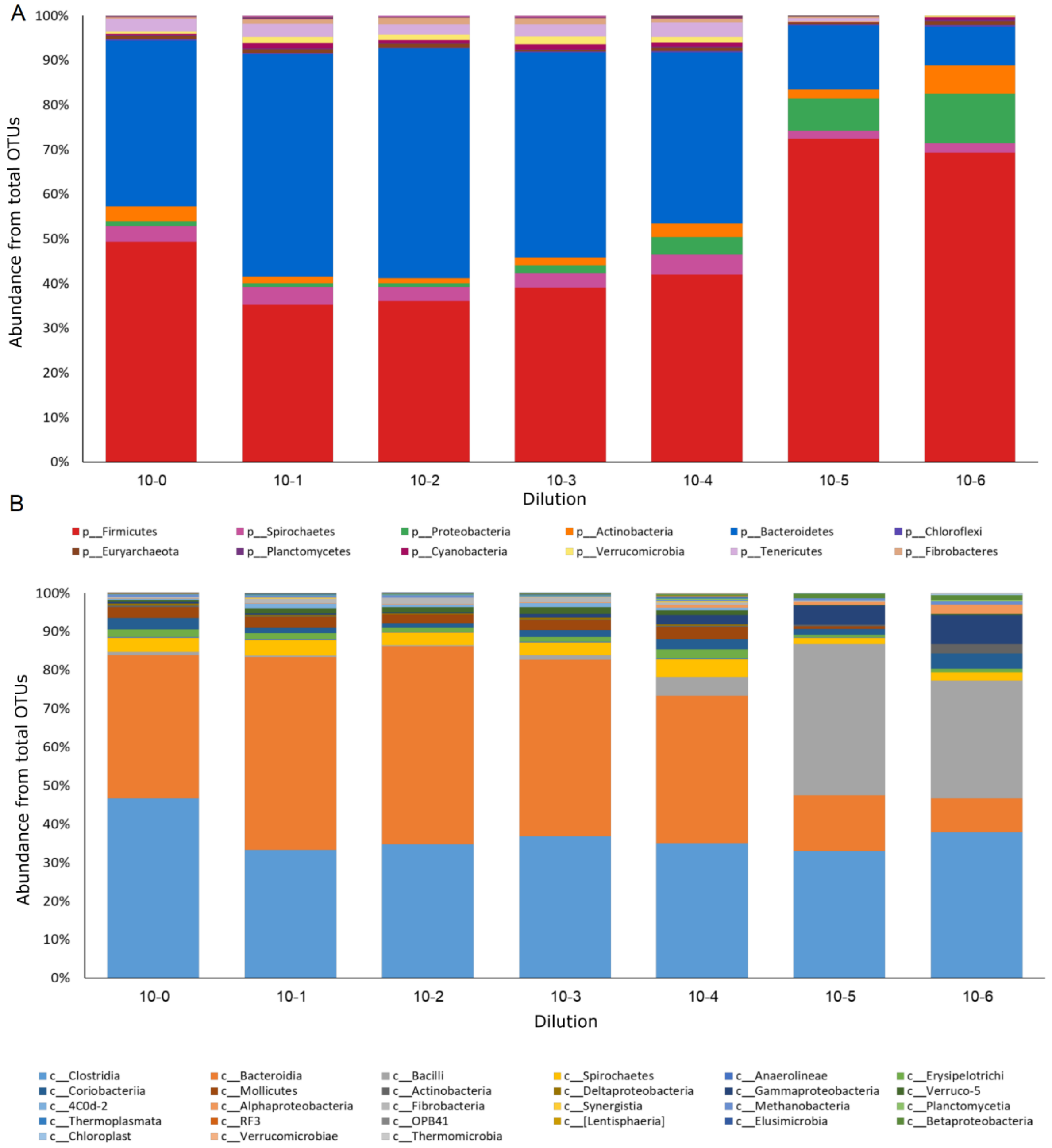

SI Figure 1 Phylogenetic composition of the original rumen sample and its dilutions used for the cultivation in this study according to the percentage of reads in each sample that were annotated on the level of the phylum (**A**) and the class (**B**), respectively.

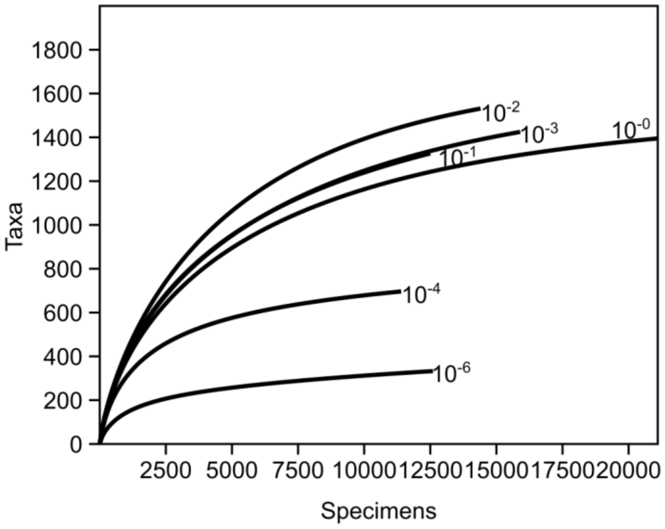

SI Figure 2 Sample rarefaction curves. The number of OTUs (x – axis) depending on the number of reads sampled (y – axis) is illustrated to visualize the sequencing depth.

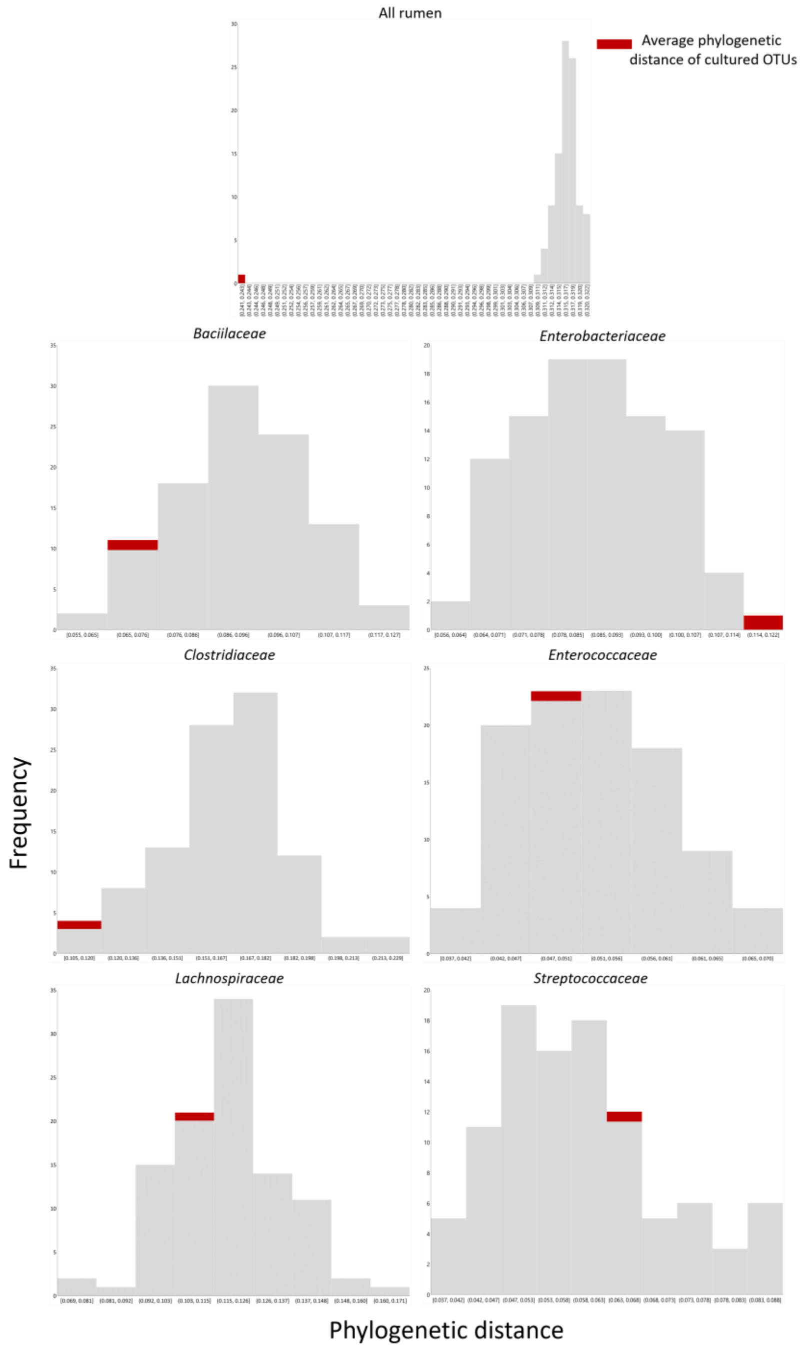

SI Figure 3 Comparison of the phylogenetic distances between OTUs growing on the plates and OTUs detected in the original rumen sample. The mean phylogenetic distances between OTUs that grew on the plates was calculated and compared to the distribution of phylogenetic distances from the OTUs of the original rumen sample. The distribution of distances from the rumen OTUs is illustrated as a histogram. The position of the average phylogenetic distance between OTUs of the cultured reference group within this rumen phylogenetic distance distribution is illustrated as red bar. The distribution of phylogenetic distances was calculated from a rumen OTU subset picked by random in a sample size depending on the cultured OTU reference group. Permutation = 100. The following numbers of rumen OTUs were picked for each category (according to the x axis): All = 1012; Bacillaceae = 6, Clostridiaceae = 6, Enterococcaceae = 6, Lachnospiraceae = 6, Streptococcaceae = 6, Enterobacteriaceae = 6.

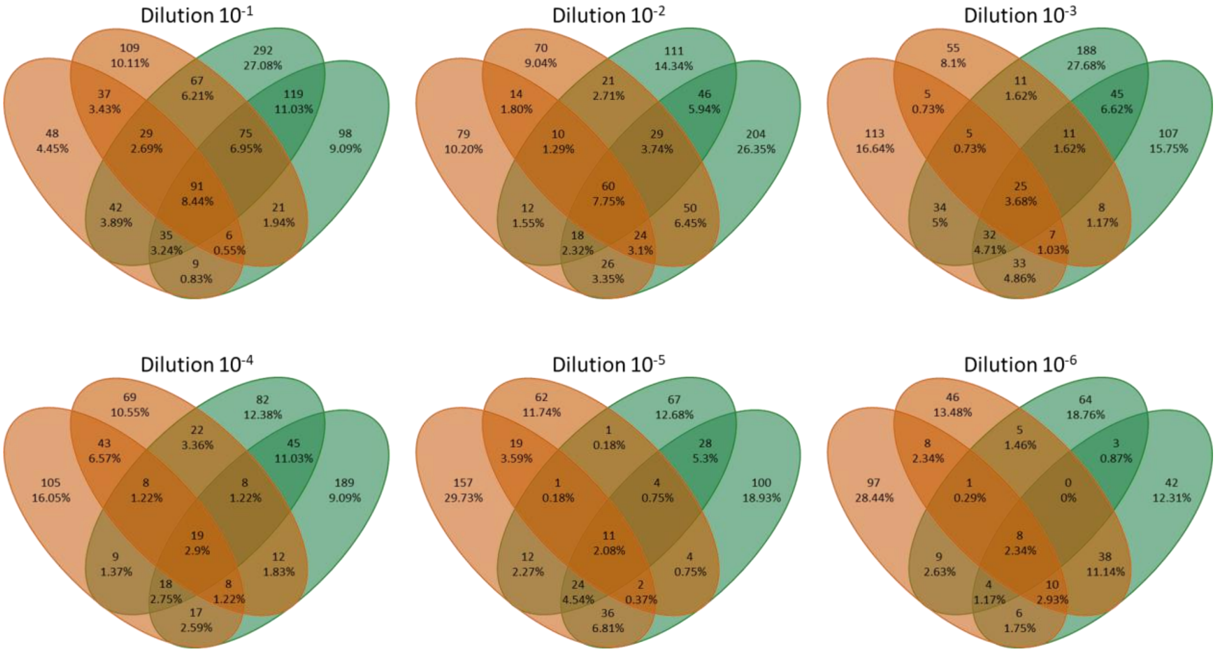

SI Figure 4 Shared OTUs between plates per dilution. The number of OTUs found on both the plates and the respective dilution of the rumen sample are presented as a Venn diagram. The percentages were calculated from the sum of OTUs per dilution. Colours represent medium type: Orange- defined medium, Green- undefined medium. In each dilution and for each medium, respectively, there are two circles representing the data for the two technical replicates.
